# Coupling CFD and Machine Learning to Assess Flow Properties in Porous Scaffolds for Tissue Engineering

**DOI:** 10.3390/mi16101098

**Published:** 2025-09-27

**Authors:** Jennifer Rodríguez-Guerra, Pedro González-Mederos, Nicolás Amigo

**Affiliations:** 1 Departamento de Biomateriales Cerámicos y Metálicos, Centro de Biomateriales, Universidad de La Habana, Ave. Universidad s/n entre G y Ronda, Vedado, La Habana 10400, Cuba; jennifer.rodriguez.guerra98@gmail.com (J.R.-G.); pedroernestog97@gmail.com (P.G.-M.); 2 Departamento de Física, Facultad de Ciencias Naturales, Matemática y del Medio Ambiente, Universidad Tecnológica Metropolitana, Las Palmeras 3360, Ñuñoa 7800003, Santiago, Chile

**Keywords:** permeability, wall shear stress, machine learning, statistics, computational fluid dynamics

## Abstract

Computational fluid dynamics and machine learning (ML) models are employed to investigate the relationships between scaffold topology and key flow parameters, including permeability (*k*), average wall shear stress ($WSS_{a}$), and the 25th and 75th percentiles of WSS. Statistical analysis showed that $WSS_{a}$ values are consistent with those found in common scaffold architectures, while percentile-based WSS properties provided insight into shear environments relevant for bone and cartilage differentiation. No significant effect of pore shape was observed on *k* and $WSS_{a}$. Correlation analysis revealed that *k* was positively associated with topological features of the scaffold, whereas WSS metrics were negatively correlated with these properties. ML models trained on six topological and flow inputs achieved a performance of $R^{2}$ above 0.9 for predicting *k* and $WSS_{a}$, demonstrating strong predictive capability based on the topology. Their performance decreased for $WSS_{25\%}$ and $WSS_{75\%}$, reflecting the difficulty in capturing more specific shear events. These findings highlight the potential of ML to guide scaffold design by linking topology to flow conditions critical for osteogenesis.

## 1. Introduction

Tissue engineering is an emerging field in biomedical engineering that leverages both life sciences and engineering to repair, maintain, or restore tissue functions, involving biomaterials, cells, and growth factors, among others [1,2,3]. This approach has gained increasing interest in repairing bone defects, as scaffolds can be used to mimic the bone matrix structure, providing an environment for cell attachment, growth, and tissue development. While scaffolds provide mechanical stability to the tissues, they also should exhibit a porous structure to promote osteoconductivity and osteoinductivity, resulting in additional challenges [4,5,6].

Previous studies have shown that the permeability of the scaffold and the wall shear stress (WSS) exerted by fluid flows play a relevant role in osteogenesis, that is, cell adhesion and bone regeneration. However, both properties are influenced by the pore topology, hindering the evaluation of the performance of the scaffold [7]. Computational fluid dynamics (CFD) has emerged as a power computational technique to investigate in detail the fluid flow in scaffolds, such as nutrient diffusion [8], permeability and porosity relantionships [9], and WSS distribution on scaffold surfaces [10], among others. Additionally, Rabiatul et al. explored the relationship between permeability and porosity for different trabecular bone orientations. The authors observed a good correlation, although it was dependent on structural orientation [11]. Fu et al. evaluated different scaffold models for trabecular bones, indicating that bone regeneration was affected by the topology [10]. Ali et al. conducted additional studies focusing on the characterization of permeability and WSS in scaffolds. They found that structures with minimal variation in channel size exhibited increased permeability. Nevertheless, no clear correlation was observed between regular topologies and WSS [9]. Suffo & López-Marín conducted a combined fluid–structure interaction study to evaluate the mechanical performance of scaffolds subjected to fluid flows at high Reynolds numbers, comparing various turbulence models using inlet velocities of 2 m/s [12]. More recently, Manescu et al. performed simulations on Mg-based alloys, known for their biodegradable capability, showing that both the mechanical performance and permeability of these structures fell within the biological range of trabecular bone [13]. Kong et al. explored various scaffold shapes, evaluating flow rates and WSS across different regions of the scaffolds to assess their capacity for efficient nutrient exchange, among other factors [14]. Additional CFD studies have investigated heterogeneous porous scaffolds with Voronoi-based architectures, showing that variations in porosity influence the internal velocity and pressure distributions, as well as permeability, affecting their potential for bone tissue engineering [15].

While numerous studies have explored the flow effects on scaffolds, there is a lack of systematic investigations addressing the relationships between scaffold topology and both permeability and WSS. As reported by Kong et al., biological phenomena such as bone tissue, cartilage tissue, and fibrous tissue differentiation are associated with distinct WSS ranges [14]. This highlights the importance of systematically examining the aforementioned relationships. Here, machine learning (ML) emerges as a formidable approach to establish predictive models for permeability and WSS based on topological properties. Several previous studies have implemented supervised learning models to predict outcomes such as the rupture risk of cerebral aneurysms [16,17], cardiovascular diseases [18,19], and the transmission of airborne infections [20,21]. These studies underscore the predictive power of ML, suggesting its strong potential for advancing scaffold design and optimization.

In this study, the permeability and WSS of three different types of scaffolds with regular structures were investigated using CFD simulations. Scaffolds with circular, cubical, and hexagonal pores were constructed, with diameters ranging from 0.2 to 0.5 mm, giving a total of 12 different topologies. Supervised machine learning models were implemented to predict the permeability and WSS on the scaffolds based on the inlet velocity, pore surface, porosity, and structure type, resulting in models that captured the underlying relationships. This integrated computational approach provides an efficient framework for evaluating scaffold designs, paving the way for data-driven optimization in tissue engineering.

## 2. Materials and Methods

### 2.1. Scaffold Construction

**Figure 1:**
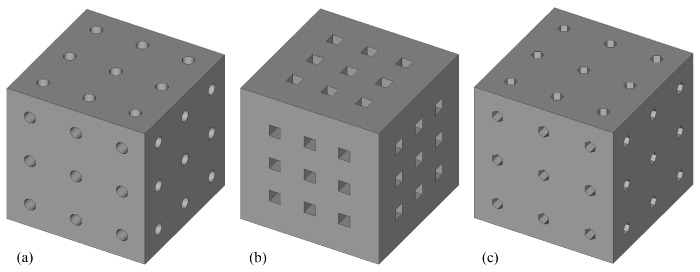
The 0.5 mm diameter pores in (**a**) circular, (**b**) cubical, and (**c**) hexagonal scaffolds, with nine pores on the front face.

Cubic scaffolds with 6 mm edges and regular pore distributions were constructed in FreeCAD 1.0 software. Three pore shapes were considered, namely circular, square, and hexagonal. For each shape, four pore diameters (0.2–0.5 mm) and three pore numbers on the front face (4, 9, and 16) were modeled, resulting in a total of 36 scaffold configurations. As an example, Figure 1 illustrates scaffolds with 0.5 mm diameter pores in circular, square, and hexagonal topologies. The scaffolds were placed inside a box with dimensions of 6.2×6.2×25 mm^3^ for the CFD simulations, as described below.

### 2.2. Boundary Conditions and Numerical Schemes

**Figure 2:**
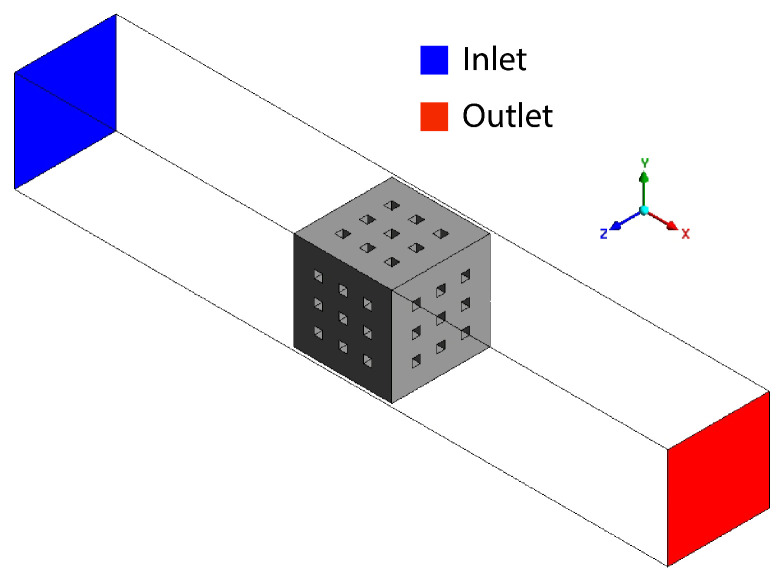
Simulation box and a scaffold with cubical pores. The inlet and outlet faces are also displayed.

The incompressible and laminar Navier–Stokes equations were used to model the fluid flow through the scaffolds. No-slip boundary conditions were applied to the box and scaffold surfaces, with a constant inlet velocity and zero outlet pressure. A scheme is shown in Figure 2. Four inlet velocities were considered, namely 0.1, 0.2, 0.3, and 0.4 mm/s, as they correspond to typical velocities found in trabecular bones [22,23]. The fluid was modeled as blood, with a constant density of 1065 kg/m^3^, using the non-Newtonian Carreau blood model with parameters λ=39.418 s, n=0.48, $\mu_{\mathrm{in}f}=0.00345$ Pa·s, and $\mu_{0}=0.0161$ Pa·s, as reported in a previous study [24]. The value of $\mu_{\mathrm{in}f}$ has been typically used as the constant dynamic viscosity for blood in Newtonian models [25,26,27]. A constant inlet velocity was employed as blood flow, and blood volume in trabecular bone does not vary significantly with the cardiac cycle [28,29]. The equations were solved using the SIMPLE algorithm with second-order schemes for velocity and time. Simulations were performed for a total of 200 steps with a time step size of 0.005 s. The residual criterion of convergence for the continuity and velocity components were set to $10^{-6}$. All CFD simulations were carried out using Ansys Fluent 2025 R1.

### 2.3. Meshing

**Figure 3:**
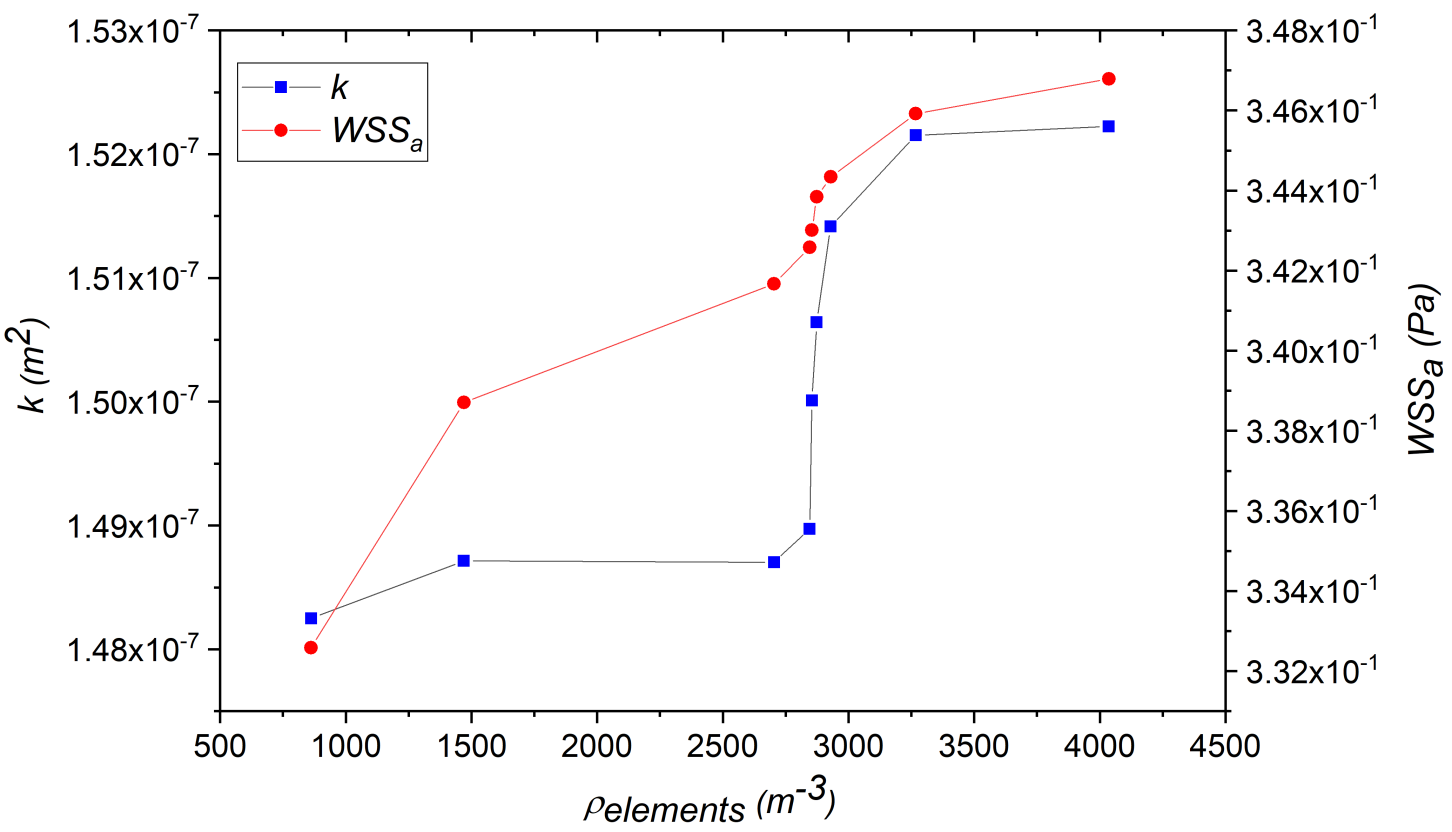
Average WSS and permeability (*k*) for the scaffold with 0.2 mm circular pore diameter.

All samples consisting of the scaffold and surrounding box were meshed with tetrahedral elements using Ansys meshing. To inspect the effect of mesh refinement, different mesh densities ($\rho_{elements}$) were analyzed, ranging from 862 to 4036 $m^{-3}$. The average WSS ($WSS_{a}$) over the scaffold surface and the permeability (*k*) were calculated using the scaffold with a 0.2 mm circular pore diameter. The permeability was calculated by transforming Darcy’s law using the flow rate Q=Av, with *A* is the scaffold area and *v* the inlet velocity, leading to

(1) $$k=\frac{v\mu L}{\Delta P},$$

where *v* is the inlet velocity, μ is the fluid viscosity within the scaffold, *L* is the length of the scaffold, and ΔP is the pressure drop across the scaffold [10]. As observed in Figure 3, both *k* and $WSS_{a}$ increased with the mesh density and reached relatively stable values for densities above 3000 $m^{-3}$. Nevertheless, the total variations in both quantities between the lowest and highest mesh densities were below 4.5%, indicating limited sensitivity in this range. Based on these observations, a mesh density of ∼ 3000 m^−3^ was adopted for all simulations in this study. We note here that mesh sensitivity analysis was also conducted on the 0.3 mm, 0.4 mm, and 0.5 mm circular pore scaffolds, resulting in no statistically significant differences for the larger densities discussed here.

### 2.4. Machine Learning Methods

A total of 144 cases were obtained, corresponding to the three pore shapes, four pore diameters (*D*), three different numbers of pores in the front face (*N*), and four inlet velocities (*v*). Additional topological descriptors were also calculated, including the porosity degree (ϕ), total scaffold surface ($S_{t}$), and pore surface ($S_{p}$). These quantities were considered as input properties. Flow parameters were then calculated to evaluate the scaffold capability for osteogenesis, including permeability (*k*), average WSS ($WSS_{a}$), first-quartile WSS ($WWS_{25\%}$), and third-quartile WSS ($WWS_{75\%}$). These four properties served as response variables. Regression models were implemented to predict *k*, $WSS_{a}$, $WWS_{25\%}$, and $WWS_{75\%}$ based on the input properties. Permeability and average WSS were chosen, as they are commonly employed to evaluate the scaffold capability for osteogenesis, while $WWS_{25\%}$ and $WWS_{75\%}$ were chosen because they provide insights into the localized shear stress environment, which is critical for guiding tissue differentiation processes such as bone and cartilage formation. All variables were standardized for comparison purposes using

(2) $$z_{i}^{s}=\frac{z_{i}-\mu }{\sigma },$$

where $z_{i}$ is the original variable, μ is its mean, and σ is its standard deviation. Data handling and transformation were conducted with the Pandas library in Python 3.13.5 [30].

Linear regression, support vector regression (SVR), K-nearest neighbor (KNN) regression, and random forest (RF) regression were explored to predict the flow properties through the scaffolds [31,32,33]. Table 1 shows a summary of the hyperparameters used for each case. The models were trained using 10-fold cross-validation [34], and their performance was evaluated by averaging the coefficient of determination ($R^{2}$) across all cases. $R^{2}$ is defined as

(3) $$R^{2}=1-\frac{\sum_{i=1}^{n}{\left(y_{a}-y_{p}\right)}^{2}}{\sum_{i=1}^{n}{\left(y_{a}-\bar{y}\right)}^{2}},$$

where *n* is the total number of observations (144 in this work), $y_{a}$ is the actual value obtained from the CFD simulations, $y_{p}$ is the predicted value by the regression model, and $\bar{y}$ is the mean of $y_{a}$. All calculations were carried out using the Scikit-learn library in Python [35].

## 3. Results

### 3.1. Statistical Analysis

Statistical exploration of the *k*, WSS, $WSS_{m}$, $WWS_{25\%}$, and $WWS_{75\%}$ variables was conducted to gain insights into their behavior. Table 2 shows the mean ($\bar{x}$), standard deviation (*s*), minimum, and maximum values for each variable. The values for *k* were slightly higher compared to previous studies with similar pore diameters [10,13] but still within the range observed in bulk cancerous bone [36,37] and solid gyroid scaffolds [38]. To further decrease the permeability, the models should be scaled down while maintaining the porosity degree [39]. For $WSS_{a}$, the values range from 0.005 Pa to 0.416 Pa, with a mean of 0.148 Pa, in agreement with those reported for other topologies such as lattice diamond, octet, and gyroid [9,14]. The $WWS_{25\%}$ and $WWS_{75\%}$ were also considered, as they provide insights into the differentiation processes occurring within the scaffold [14,40]. The mean value for $WWS_{25\%}$ is 0.004 Pa, which is beneficial to bone tissue differentiation, while the mean value for $WWS_{75\%}$ is 0.276, which is beneficial to cartilage tissue differentiation.

The relationship between *k* and $WSS_{a}$ and the pore shape was also examined. Table 3 presents the average value for each quantity. Although the values are relatively similar across the groups, a single statistical measure is not enough to determine whether pore shape has a significant effect on the flow properties. To address this matter, the non-parametric Kruskal–Wallis test was performed, yielding *p*-values of 0.052 for *k* and 0.11 for $WSS_{a}$. These results indicate that there is no statistically significant difference between the groups [41,42] at the conventional 0.05 level, suggesting that pore shape does not have a relevant effect on the permeability and average WSS.

### 3.2. Correlation Analysis

**Figure 4:**
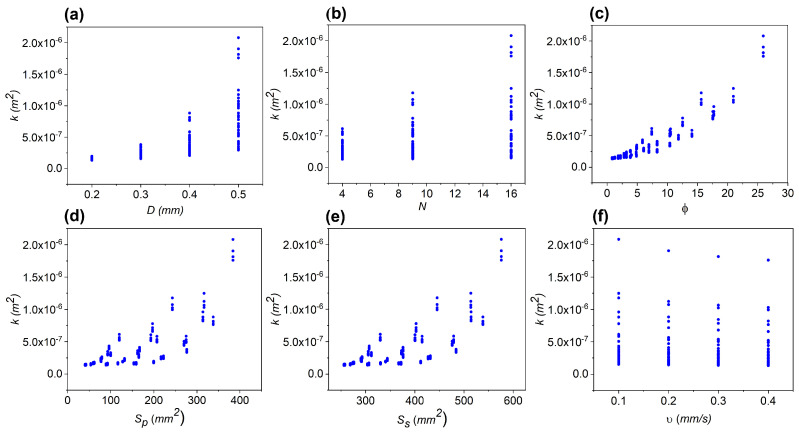
Relationships between the permeability (*k*) and six different input properties. Increasing trends are observed for *k* with *D*, *N*, ϕ, $S_{p}$, and $S_{s}$ as shown in panels (**a**–**e**), while a slightly decreasing trend is distinguished for *k* with *v* in some cases as shown in panel (**f**).

The relationships between *k* and the input properties *D*, *N*, *v*, ϕ, $S_{p}$, and $S_{t}$ are shown in Figure 4, where each data point corresponds to an individual simulation. Increasing trends are observed in all cases except for *v*, where a slight decreasing variation occurs. As reported in previous studies, larger pore diameters, surfaces, and porosity degrees promote enhanced flow through the scaffold. In the case of *v*, the slight decreasing trend observed for a few cases may be attributed to minor deviations from Darcy’s law.

**Figure 5:**
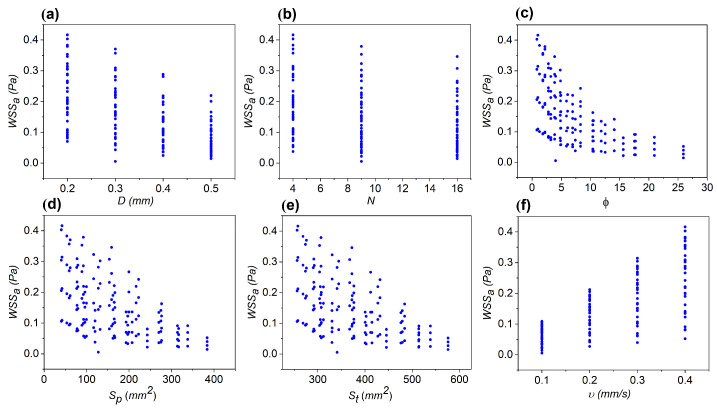
Relationships between the average WSS ($WSS_{a}$) and six different input properties. Decreasing trends are observed for $WSS_{a}$ with *D*, *N*, ϕ, $S_{p}$, and $S_{s}$ as shown in panels (**a**–**e**), while the opposite behavior occurs for $WSS_{a}$ with *v* (**f**).

When inspecting the behavior of $WSS_{a}$, decreasing trends are observed for all cases except for *v*, as shown in Figure 5. Previous studies have attributed the inverse relationship between porosity and WSS to flow redistribution and energy dissipation within the porous structure. A higher number of pores results in more interconnected channels that facilitate fluid flow, causing the flow to be more uniformly distributed throughout the scaffold surface. This redistribution reduces the concentration of high velocities near the walls, decreasing velocity gradients and, consequently, the WSS [9,43,44]. Additionally, the increasing trend of $WSS_{a}$ with *v* corresponds to the enhanced shear forces exerted by the fluid as velocity rises, leading to greater WSS on the scaffold surfaces.

The Spearman’s correlation coefficient (ρ) was calculated to evaluate the degree of correlation between the different properties considered in this study. ρ is given by

(4) $$\rho =1-\frac{6\sum_{i=1}^{n}d_{i}^{2}}{n(n^{2}-1)},$$

where $d_{i}$ represents the difference between the two ranks of the pair of properties and *n* is the number of observations, which is 144 in this work. A positive ρ indicates a positive correlation between the two pair of variables, while a negative ρ corresponds to a negative correlation. A good correlation is inferred when 0.6<|ρ|<0.8 and a strong correlation when |ρ|>0.8.

**Figure 6:**
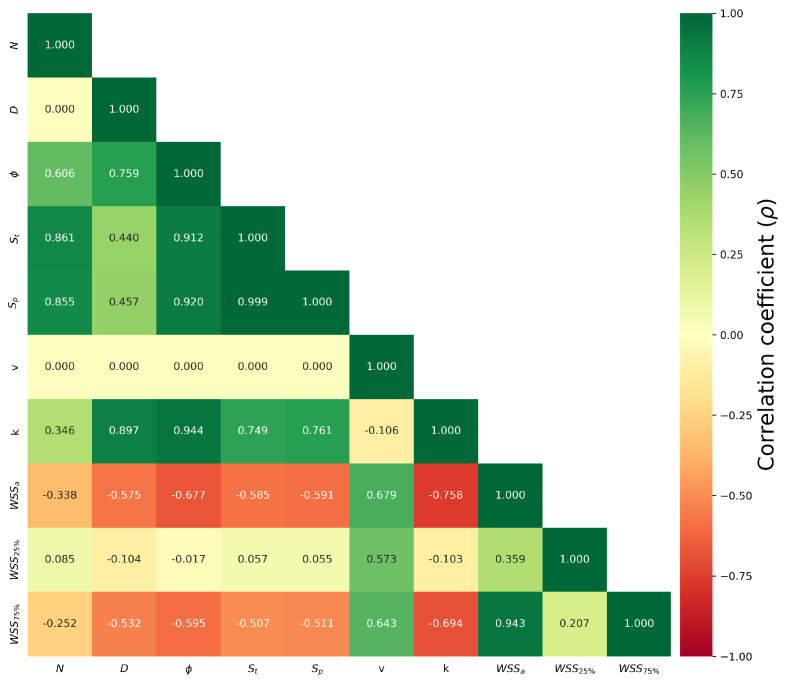
Spearman’s correlation analysis among the properties considered in this study.

The resulting values are shown in Figure 6. Positive correlations are found between topological properties such as the pore number, porosity degree, pore surface, and total scaffold surface, while no correlation is observed for *v* with these properties. This is expected since the inlet velocity is set as a boundary condition in the CFD simulation, whereas the topological properties are intrinsic to the scaffold design. An inspection of the permeability reveals that relevant positive correlations exist with the pore diameter, porosity degree, total scaffold surface, and pore surface, reflecting the enhanced flow through the scaffold. On the other hand, negative correlations are observed for $WSS_{a}$, $WSS_{25\%}$, and $WSS_{75\%}$ with the topological properties, consistent with the reduced concentration of high velocities at the scaffold surface discussed previously.

### 3.3. Predictive Models

Seven quantities were initially considered as input properties to predict flow parameters on the scaffolds:, namely the pore shape, pore diameter (*D*), pore number in the front face (*N*), inlet velocity (*v*), porosity degree (ϕ), pore surface ($S_{p}$), and total scaffold surface ($S_{t}$). However, based on the exploratory statistical analysis, pore shape was discarded, as it showed no correlation with permeability or average WSS. Consequently, the predictive models were constructed as follows:

(5) $$y=f(D,N,v,\phi ,S_{p},S_{t}),$$

where *y* is the predicted flow parameter, representing either *k*, $WSS_{a}$, $WSS_{25\%}$, or $WSS_{75\%}$, and *f* is the regression model

**Figure 7:**
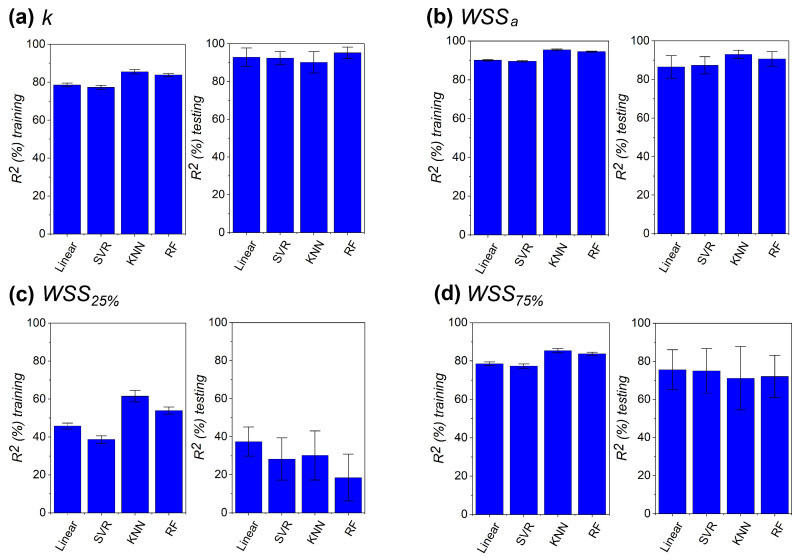
Average coefficient of determination ($R^{2}$) for the training and testing sets delivered by the four ML models. Standard deviations are denoted by the black bars. Remarkable performance is observed for *k*, $WSS_{a}$, and $WSS_{75\%}$ in panels (**a**,**b**,**d**), while a poor predictive capability is observed for $WSS_{25\%}$ in panel (**c**).

In the 10-fold cross-validation process, the coefficient of determination ($R^{2}$) was calculated for each model, yielding ten values per case. The mean $R^{2}$ was then computed for both the training and testing sets, as shown in Figure 7, where black bars represent the standard deviations. The models exhibited excellent performance for permeability, with predictive capability exceeding 90%. Similar results were obtained for $WSS_{a}$, indicating that the flow properties commonly used to evaluate scaffold suitability for osteogenesis can be predicted from topological parameters. In contrast, model performance decreased significantly for $WSS_{25\%}$ and $WSS_{75\%}$, with larger standard deviations observed in the testing sets. This effect was particularly pronounced for $WSS_{25\%}$, suggesting that predicting localized low-shear and high-shear regions is inherently more challenging due to the higher sensitivity required to capture subtle flow disturbances. Interestingly, the performance across the different ML models is very similar, indicating that the flow parameters are strongly governed by the underlying scaffold topology rather than the choice of the regression algorithm. This robustness suggests that relatively simple models, such as linear regression, can achieve predictive accuracy comparable to more complex approaches, in contrast to other studies where more sophisticated methods, such as RF, achieved superior performance [45,46,47,48]. The dataset generated in this study can be found in the Supplementary Materials.

## 4. Conclusions

This study combined computational fluid dynamics and machine learning models to investigate the influence of scaffold topology on flow-related properties, including permeability (*k*), average wall shear stress ($WSS_{a}$), and the 25th and 75th percentiles of WSS. The analysis demonstrated that topological parameters such as pore diameter, porosity, and surface areas strongly influence *k* and WSS, while pore shape had no statistically significant effect on *k* or $WSS_{a}$. Thus, the effect of shape appeared to be much weaker compared to diameter. The negative correlations between WSS measures and topological parameters reflect the effect of porosity in redistributing flow and reducing shear concentrations. Additionally, this study considered non-Newtonian effects through a Carreau fluid model, revealing differences in magnitudes compared to Newtonian assumptions. This highlights the importance of fluid rheology when modeling the scaffold performance under physiological conditions, a matter that should be further explored in future studies.

Machine learning models trained on topological properties achieved high predictive accuracy for *k* and $WSS_{a}$, demonstrating their capability to predict relevant flow properties of the scaffold for osteogenesis. However, the models delivered reduced performance for predicting $WSS_{25\%}$ and $WSS_{75\%}$, indicating that localized shear extremes are more sensitive to subtle geometric and flow variations.

Future studies should extend the analysis to a wider range of scaffold architectures, investigate transient and pulsatile flows and mechanical performance through fluid–structure interaction and non-regular topological structures, and incorporate experimentally measured flow data to further validate the predictive capability of ML approaches. Additionally, dimensionality reduction techniques should be explored to address input variable correlations, reduce model complexity, and further mitigate overfitting. The integration of advanced topology descriptors, realistic boundary conditions, and refined rheological models will enhance the ability to design scaffolds optimized for specific tissue engineering applications.

## Figures and Tables

**Table 1:** Hyperparameters used for each machine learning model.

Model	Hyperparameters
Linear Regression (LR)	Tolerance: $10^{-6}$
Support Vector Regression (SVR)	Kernel: Linear
	Gamma: scale
	Tolerance: $10^{-3}$
K-nearest Neighbor Regression (KNN)	Number of neighbors: 4
	Weights: Uniform
Random Forest (RN)	Number of estimators: 500
	Criterion: Squared error
	Max depth: 3

**Table 2:** Descriptive statistics for the flow parameters.

Property	$\bar{x}$	*s*	Min	Max
*k* (m^2^) $\left[\times 10^{-7}\right]$	4.111	3.620	1.330	20.81
$WSS_{a}$ (Pa)	0.148	0.096	0.005	0.416
$WSS_{25\%}$ (Pa)	0.004	0.003	0.001	0.013
$WSS_{75\%}$ (Pa)	0.276	0.187	0.022	0.872

**Table 3:** Mean and standard deviations for *k* and $WSS_{a}$ according to the pore shape.

Pore Shape	*k* (m^2^)	${\mathrm{WSS}}_{a}$ (Pa)
Circular	$3.745\pm 2.824\times 10^{-7}$	0.1627±0.102
Square	$5.404\pm 2.965\times 10^{-7}$	0.1253±0.088
Hexagonal	$3.185\pm 2.131\times 10^{-7}$	0.1564±0.097

## Data Availability

The original contributions presented in this study are included in the Supplementary Materials.

## Supplementary Materials

The following supporting information can be downloaded at: https://www.mdpi.com/article/10.3390/mi16101098/s1.
